# miPrimer: an empirical-based qPCR primer design method for small noncoding microRNA

**DOI:** 10.1261/rna.061150.117

**Published:** 2018-03

**Authors:** Shih-Ting Kang, Yi-Shan Hsieh, Chi-Ting Feng, Yu-Ting Chen, Pok Eric Yang, Wei-Ming Chen

**Affiliations:** Quark Biosciences, Zhubei, Hsinchu, 30261, Taiwan

**Keywords:** microRNA, primer design strategies, primer dimerization, primer specificity, real-time PCR, qPCR efficiency

## Abstract

MicroRNAs (miRNAs) are 18–25 nucleotides (nt) of highly conserved, noncoding RNAs involved in gene regulation. Because of miRNAs’ short length, the design of miRNA primers for PCR amplification remains a significant challenge. Adding to the challenge are miRNAs similar in sequence and miRNA family members that often only differ in sequences by 1 nt. Here, we describe a novel empirical-based method, miPrimer, which greatly reduces primer dimerization and increases primer specificity by factoring various intrinsic primer properties and employing four primer design strategies. The resulting primer pairs displayed an acceptable qPCR efficiency of between 90% and 110%. When tested on miRNA families, miPrimer-designed primers are capable of discriminating among members of miRNA families, as validated by qPCR assays using Quark Biosciences’ platform. Of the 120 miRNA primer pairs tested, 95.6% and 93.3% were successful in amplifying specifically non-family and family miRNA members, respectively, after only one design trial. In summary, miPrimer provides a cost-effective and valuable tool for designing miRNA primers.

## INTRODUCTION

miRNAs, short endogenous noncoding RNAs consisting of 18–25 nucleotides (nt), are involved in post-transcriptional regulation by targeting the 3′ UTR of mRNA for mRNA degradation or translation inhibition (Lee et al. 1993; Wightman et al. 1993; Reinhart et al. 2000; Bartel 2009). First identified in *Caenorhabditis elegans*, they function to regulate the development timing in larval stage (Lee et al. 1993; Wightman et al. 1993). Subsequently, over 35,828 mature miRNAs were found in 223 species, including plants, animals, and viruses (http://www.mirbase.org/).

miRNAs are key regulators of gene expression networks, controlling diverse biological processes including proliferation, differentiation, apoptosis, metabolism, development, and host−pathogen interactions (Bueno et al. 2008; Slaby et al. 2009; Small and Olson 2011). Furthermore, miRNAs have been demonstrated to be involved in cancer development (Calin and Croce 2006; Esquela-Kerscher and Slack 2006; Manikandan et al. 2008; Zhang et al. 2014; Ohtsuka et al. 2015) and the progression of other diseases (Lu et al. 2008; Jiang et al. 2009; Li and Kowdley 2012; Mendell and Olson 2012).

The implications of miRNAs in cancer and other diseases have led to significant efforts and resources divested in the understanding of miRNA expression. To date, many methods, such as small RNA sequencing, northern blot analysis, in situ hybridization, nanoparticle-based methods, miRNA qPCR assays, and miRNA microarrays, are routinely used in the detection and expression analysis of miRNAs (Catuogno et al. 2011; Tian et al. 2015). Among these methods, qPCR assay is one of the most widely used measurement methods for miRNA quantification and expression profiling because of its sensitivity, convenience, and short experimental time. Several primer and probe design methods have been developed for qPCR-based miRNA quantification, such as the stem–loop RT-qPCR method with a hydrolysis probe (Chen et al. 2005; Feng et al. 2009), SYBR Green RT-qPCR method with LNA primers (Raymond et al. 2005), and poly(A)-tailed universal reverse transcription-based method with a specific forward primer and a universal reverse primer or two specific forward and reverse primers (Fu et al. 2006; Balcells et al. 2011). Because mature miRNA are short, sometimes between 18 and 25 nt long, one of the obstacles in a miRNA qPCR assay is to design highly specific primers with a *T*_m_ of 59°C–60°C and acceptable PCR efficiency. While hydrolysis probes and LNA primers both met the criteria, they are expensive. Balcells et al. (2011) provides a cost-effective method using two specific primers, with increased *T*_m_ and specificity by adding a 5′-tail to each primer. In addition, the authors have shown that the method has a higher amplification efficiency than LNA primers, and could successfully discriminate closely related miRNAs as well as LNA primers. However, current poly(A)-tailed universal reverse transcription-based methods do not provide a systematic way of designing primers to distinguish between members of miRNA families.

In the present study, we established an algorithm called miPrimer, which significantly improves the way primers are designed in the poly(A)-tailed universal reverse transcription-based method. Using Quark Biosciences’ platform (Chang Y, Wei CW, Pan CC, Chiou CF, Chang CH, Hsieh YF, Lee CH, Huang JW, Zheng XY, Chiu CY, et al., in prep.), we have demonstrated that the design method produces primers that are capable of discriminating closely related miRNAs as well as miRNA family members with multiple- or single-nucleotide difference(s). To our knowledge, miPrimer is the first and only empirical primer design method that provides research scientists a cost-effective, systematic way of designing miRNA primers with a high success rate.

## RESULTS

### Achieving excellent qPCR efficiency with miPrimer

**FIGURE 1.**
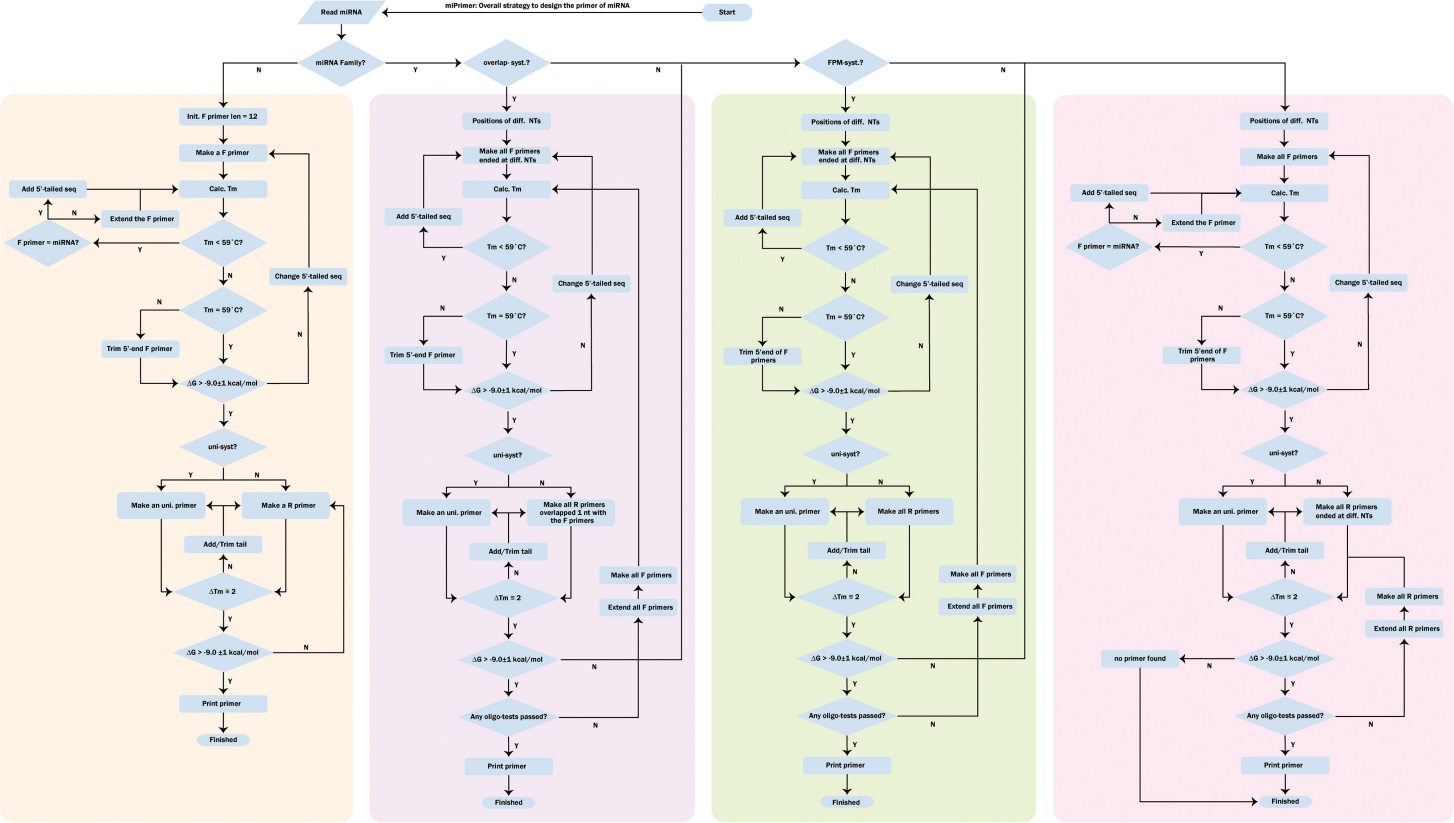
Framework of miPrimer's design strategies. miPrimer includes two major systems: uni-system and specific-FR-system. The specific-FR-system contains three sequential design rules/functions, overlap, FPM (forward primer major), and RPM (reverse primer major), to reduce dimer issues and increase primer specificity. There are four design strategies highlighted in color, from *left* to *right*, uni-system, specific-FR-system^overlap^, specific-FR-system^FPM^, and specific-FR-system^RPM^. A more detailed description of when and how to utilize the strategies is transcribed in the Materials and Methods section.

**FIGURE 2.**
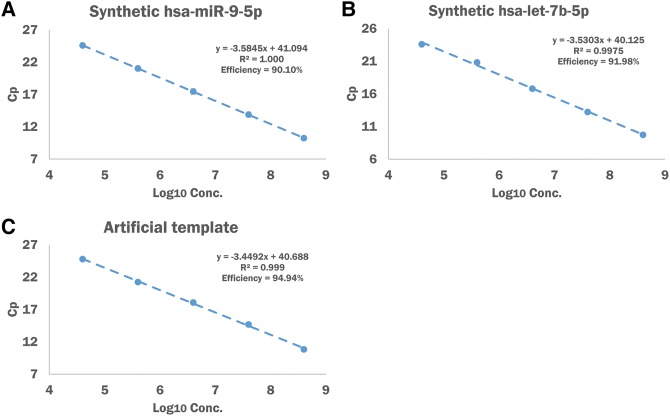
qPCR efficiency of miRNA primers designed by miPrimer. (*A*) qPCR efficiency analysis of a serially diluted oligonucleotide hsa-miR-9-5p miRNA template against hsa-miR-9-5p-F primer and universal reverse primer designed by uni-system (four 10-fold dilutions; 2500 qPCR reactions per dilution). (*B*) qPCR efficiency analysis of a serially diluted synthetic oligonucleotide hsa-let-7b against hsa-let-7b-F/R designed by specific-FR-system^FPM^ (four 10-fold dilution; 2500 qPCR reactions per dilution). (*C*) qPCR efficiency analysis of a serially diluted artificial template using the universal reverse primer and a designed forward primer (four 10-fold dilutions; 2500 qPCR reactions per dilution). Each data point on the plot represents mean Cq ± SD from three replicates.

The qPCR efficiency is impacted by a number of factors. To illustrate that the miRNA primers designed by miPrimer methodology (Fig. 1) can achieve excellent qPCR efficiency, a titration assay of four 10-fold serial dilutions was conducted with different miRNAs in various designing methods (Materials and Methods). First, a miRNA serial titration assay was performed using synthetic oligonucleotide hsa-miR-9-5p as a template, a forward primer hsa-miR-9-5p-F, and a universal reverse primer (pair of primers designed by the “uni-system,” see Fig. 1). As shown in Figure 2A, hsa-mir-9-5p demonstrated a qPCR efficiency of between 90% and 110% (*N* = 3, STD ≤ 0.41), which is considered acceptable (Robledo et al. 2014). To evaluate the qPCR efficiency of the “specific-FR-system,” oligonucleotide hsa-let-7b-5p, a member of the let-7 miRNA family, was selected as a template for the miRNA serial titration assay. The primer pairs, hsa-let-7b-5p-F and hsa-let-7b-5p-R, were designed by “specific-FR-system^overlap^” (Fig. 1). The result illustrated an acceptable qPCR efficiency of between 90% and 110% (Fig. 2B, *N* = 3, STD ≤ 0.1). To demonstrate that miPrimer can design primer pairs with acceptable qPCR efficiency for templates other than miRNA, we constructed an artificial template to be tested. The result of the serial titration assay indicated that the primer pairs for the artificial template had a qPCR efficiency of 94.95% (Fig. 2, *N* = 3, STD ≤ 0.07). We also performed titration assays for two other primer sets, has-miR-122-5p (uni-system) and has-miR-10a-5p (“specific-FR-system”). The results showed that both assays had acceptable qPCR efficiencies (Supplemental Fig. S1). Moreover, the same five pairs of primers performed on a Bio-Rad qPCR platform showed similar results (Supplemental Fig. S2), albeit with a slightly lower PCR efficiency. We believe the reason for a slight decrease in the PCR efficiency of the Bio-Rad system is due to the qPCR Master Mix, which was specific and optimized only for Quark Biosciences’ platform. Taken together, the results suggest that the miRNA primer pairs designed by either the uni-system or specific-FR-system have acceptable qPCR efficiency and can be used on various qPCR platforms.

### Discriminative identification of miRNA family using miPrimer

**FIGURE 3.**
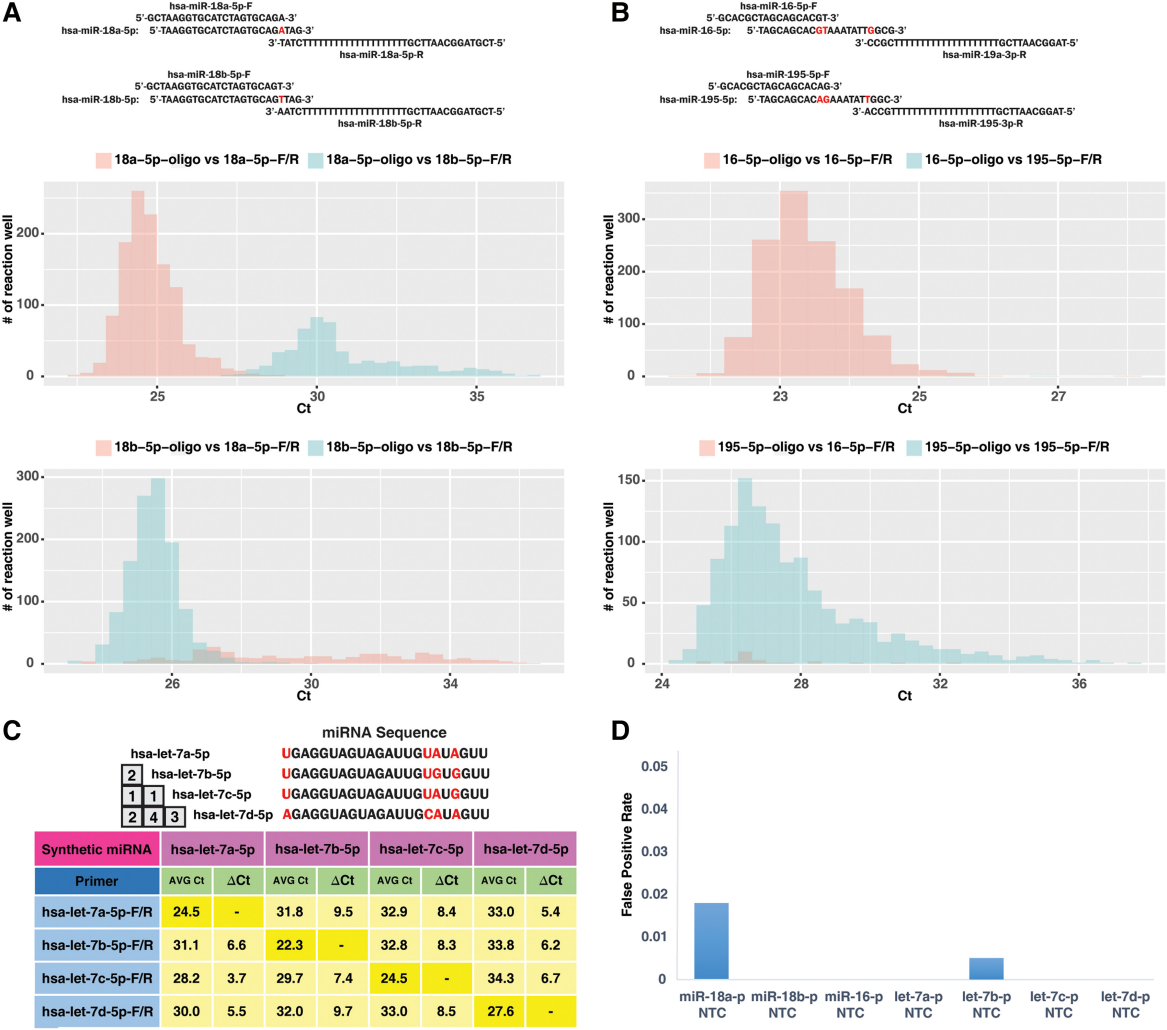
Discrimination of miRNA family with primers designed by miPrimer. (*A*) Mature miRNA sequence of hsa-miR-18 family members. Sequence differences between members are indicated in red. Primers were designed by specific-FR-system^overlap^. Discrimination of synthetic oligonucleotide hsa-miR-18 family members against homologous or heterologous miRNA primers was performed in 2500 qPCR reaction assays with Quark Biosciences’ DigiChip. Cq distributions of synthetic oligonucleotide hsa-miR-18 family members against homologous or heterologous miRNA were plotted based on the result of qPCR reactions. (*B*) Mature miRNA sequence of hsa-miR-16 family members. Sequence differences between members are indicated in red. Primers were designed by specific-FR-system^FPM^. Discrimination of synthetic oligonucleotide hsa-miR-16 family members against homologous or heterologous miRNA primers was performed in 2500 qPCR reaction assay with Quark Biosciences’ DigiChip. Cq distributions of synthetic oligonucleotide hsa-miR-16 family members against homologous or heterologous miRNA were plotted based on the result of qPCR reactions. (*C*) Mature miRNA sequence of hsa-let-7 family members. Sequence differences between members are indicated in red. (*D*) No-template control (NTC) assay for each miRNA primer was performed to estimate the false positive rate.

Discriminating between members of same miRNA family with closely related sequences in the qPCR assay represents a significant challenge. Our empirical-based method miPrimer (Fig. 1) is capable of distinguishing members of the same miRNA family by increasing primer specificity while reducing the primer dimer issue. To evaluate the capability of the primers designed by miPrimer in discriminating the miRNA family, a number of assays were designed for evaluation. The hsa-miR-18 family, consisting of two miRNAs hsa-miR-18a-5 and hsa-miR-18b-5p that differed in sequences by a single nucleotide, was used in an evaluation. A qPCR reaction was performed using synthetic hsa-miR-18 oligonucleotide, which mimics as templates, and primer pairs designed by the specific-FR-system^overlap^ (Fig. 3A). The result indicated that the synthetic mimics can be successfully discriminated by their respective primer pairs, either the forward primer hsa-miR-18a-5p-F/reverse primer hsa-miR-18a-5p-R set or the forward primer hsa-miR-18b-5p-F/reverse primer hsa-miR-18b-5p-R set (Fig. 3A). Despite the range of the Cq value (this type of range is often observed in nano-volume qPCR platforms as described in Farr et al. 2015), the experiment served the purpose of illustrating the specificity of the primer pairs. In another illustration, the primers designed by specific-FR-system^FRM^ for hsa-miR-16 family members, hsa-miR-16-5p and hsa-miR-195-5p, can distinguish synthetic hsa-miR-16 family oligonucleotide template mimics (Fig. 3B).

The hsa-let-7 family, consisting of eight miRNAs where four of the eight have almost identical sequences, creates one of the biggest challenges in primer designs. Our design process was able to produce primers that distinguish hsa-let-7 family members, achieving up to <5% cross-reactivity between one or more nucleotide mismatches (Fig. 3C). Only the hsa-let-7c miRNA assay showed 8% cross-reactivity against a synthetic hsa-let-7a-5p miRNA template (Fig. 3C). Finally, we excluded nonspecific binding by performing no-template control (NTC) experimental assays. One exception was hsa-miR-18a, which has a false positive rate close to 2% (Fig. 3D). False positive rate is calculated by P_NTC_/*N*, where P_NTC_ denotes the number of reactions in NTC assay, and *N* denotes the total number of qPCR reaction assays (in our case, 2500 reactions). In addition, to illustrate that the primers will distinguish cDNA product from miRNA synthetic RNA template, we performed reverse transcription on the template followed by qPCR analysis. As shown in Supplemental Table S2, the hsa-let-7b primer pair recognized only the cDNA synthesized from hsa-let-7b synthetic RNA template, but not other synthesized cDNA. In conclusion, the results demonstrated that miPrimer primers are capable of discriminating members of the miRNA family in qPCR reactions through the increase of primer specificity and the elimination of the primer dimer issue.

The specificity of the primers was also tested on A2058 cell line miRNAs using the Bio-Rad qPCR platform (Supplemental Table S3). Based on the melting curve analysis, we observed only a single peak for each primer set tested. This illustrates that the primer pairs work not only on synthetic templates but also on miRNA templates from real samples. Further, we added additional evidence that the primer design could work across different qPCR platforms.

### Efficiency of designing miRNA primer via miPrimer

Designing primers for miRNAs is time-consuming and costly, especially for members of miRNA family or miRNAs with similar sequences. A total of 120 miRNA primer pairs were designed by miPrimer (Table 1) and validated with synthetic oligonucleotide template mimics on Quark Biosciences’ qPCR platform (data not shown). A statistical analysis in Table 1 shows the efficiency of designing primer based on the principles of miPrimer. In our case, the less the number of times a primer pair needs to be redesigned, the more efficient the process is. As shown in the Supplemental Table S2, 95.6% of the primer pairs for 90 non-family miRNAs were successfully designed by miPrimer with the uni-system in the first trial. The remaining 4.4% of the primer pairs were redesigned utilizing the specific-FR-system to address the dimer issue (Table 1). In addition to the 90 non-family miRNAs, there were 17 miRNA families (or similar miRNA groups), consisting of 30 total miRNAs, whose primer pairs have been designed by miPrimer. Of the 30 miRNAs, only the primer pairs of two miRNAs were required to be further adjusted to include a 2-nt overlap between the forward and reverse primers (Table 1). In our current study, there were no miRNA primer designs that required the specific-FR-system^RPM^, which implied that the dimers were frequently caused by the reverse primer. In summary, miPrimer provides an efficient way of designing primers for miRNAs.

**TABLE 1.**
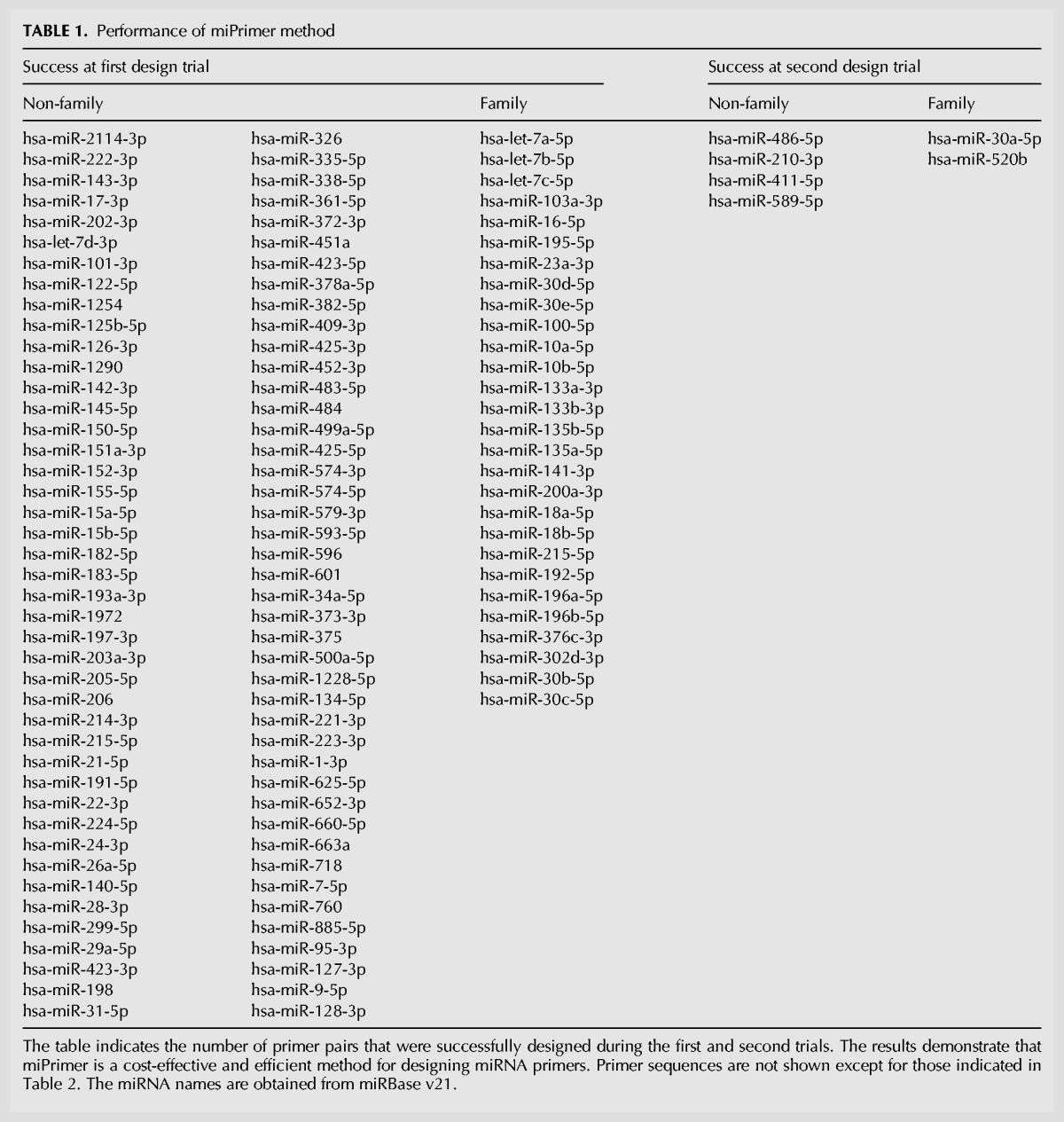
Performance of miPrimer method

## DISCUSSION

The process of designing miRNA primer, requiring both dry laboratory tools and wet laboratory validations, can be time-consuming and arduous. miPrimer, an empirical-based method, was developed by learning from several failed cases during miRNA primer design phases. Through the fine-tuning of miPrimer, we were able to come up with an effective and economical way of designing miRNA primers. In addition to reasonable qPCR efficiencies, the primer dimer issue was greatly reduced and the primers exhibited increased specificity. Of the primer pairs designed for 120 miRNAs, 95% were successful in the first trial for either non-family or family members. Overall, miPrimer is an exceptional tool for the primer design of small noncoding miRNAs.

In the process of primer design, Δ*G* is one of most critical factors used to determine the presence of dimers (Shen et al. 2010). Primers or primer pairs calculated by different algorithms showed different Δ*G* values. Calculated using OligoAnalyzer 3.1, hsa-miR-16-5p-F and hsa-miR-195-5p- had a low Δ*G* value of −10.44 and −10.44 kcal/mol, respectively. However, the Δ*G* values of these primers were considered acceptable in miPrimer (Table 2), as validated by our no-template control experimental assays (Fig. 3D). In comparison, hsa-miR-18a-5p-F and hsa-let-7b-5p-F, with extremely low Δ*G* value as calculated by miPrimer or OligoAnalyzer 3.1, exhibit false positive signals in the no-template control reactions, an indication of dimer formation. Therefore, various Δ*G* determining tools, along with miPrimer, should be used when designing primers in our case.

**TABLE 2.**
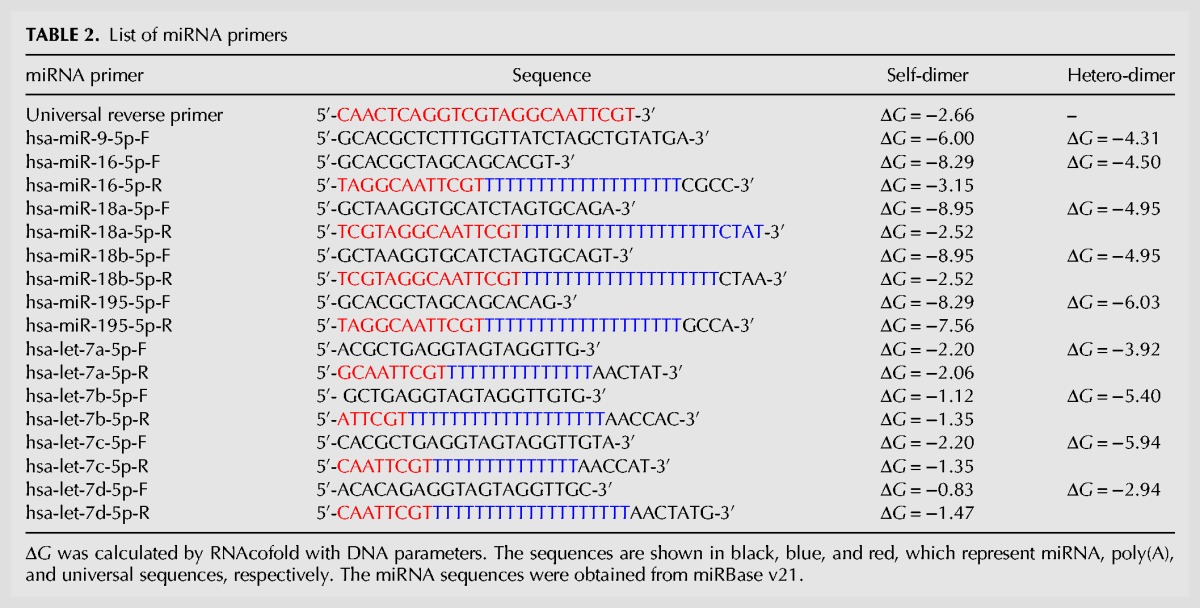
List of miRNA primers

Mestdagh et al. (2014) reported that most miRNA detection methods showed cross-reactivity between let-7 family members (hsa-let-7a-5p, hsa-let-7b-5p, hsa-let-7c-5p, and hsa-let-7d-5p), with the exception of miRCury (Exiqon's LNA primers). As an example, high cross-reactivity was observed in the hydrolysis measurement method in one of the let-7 family assays. Utilizing the design process of miPrimer and Quark Biosciences’ platform (Chang Y, Wei CW, Pan CC, Chiou CF, Chang CH, Hsieh YF, Lee CH, Huang JW, Zheng XY, Chiu CY, et al., in prep.), little or no cross-reactivity was shown between hsa-let-7 family members. Generally, a stretch of 20 dT residues was used in the specific reverse primer with an exact match to the template. If any primer pairs designed by miPrimer cannot successfully distinguish between miRNA family members with a cross-reactivity of <5%, the length of the dT in the reverse primer will be decreased to 15 dT residues to reduce cross-reactivity. For example, let-7a-5p miRNA assay exhibited high cross-reactivity to the synthetic let-7b-5p using the specific reverse primer with a stretch of 20 dT residues (data not shown). After replacing the reverse primer with 15 dT residues, the cross-reactivity of the let-7a-5p miRNA assay to the synthetic let-7b-5p was reduced to 2% (Fig. 3C). Another similar example is let-7c-5p. After replacing the reverse primer with 15 dT residues, the cross-reactivity of the let-7c-5p miRNA primers to synthetic templates of let-7a-5p and let-7b-5p was reduced to <0.4% (data not shown). Therefore, shortening the length of 20 dT residues to at least 15 dT residues in the specific reverse primer might be helpful in distinguishing between miRNA family members based on our experiences.

As illustrated from the above discussion, although miPrimer is a powerful tool, there is still room for improvement in the primer design of miRNA families. Various Δ*G* determining tools and the cross-reactivity ratio are a number of features that can be used to resolve potential issues. The primer specificity is still a key factor in separating members of miRNA families. Identifying additional features for primer design of miRNA families in order to improve the primer specificity will continue to be a very important task and can be incorporated into the miPrimer algorithm. Further, the methodology cannot distinguish isomiRs. Thus, future improvements can also be done to the methodology to help resolve the issues of isomiRs distinction.

## MATERIALS AND METHODS

### Flow schematics of primer design for miRNA

miPrimer is an empirical-based methodology comprised of two methods, uni-system and specific-FR-system, for designing primers (Fig. 1). When the sequence similarity of the miRNA of interest is <90% of that compared to other miRNAs, the uni-system is first adopted to design primer. The uni-system is preferred as the primers are easier to design and the process is cost-effective and less time-consuming. On the other hand, the specific-FR-system is used when the sequence similarity is higher than 90%, e.g., hsa-let-7 family. The specific-FR-system has three embedded functions, overlap, FPM (forward primer major), and RPM (reverse primer major), which aim to reduce primer dimer issue and increase primer specificity. As a result of these embedded functions, the probability of  discriminating between highly similar miRNAs is increased. In addition to the above-described systems, the methodology requires two sequences depending on the rules and conditions; a 5′-tailed sequence 5′-CGWTSSRCRC-3′ that will be added to forward primers, and a universal primer 5′-CAACTCAGGTCGTAGGCAATTCGT-3′ that will act as the reverse primer. Below, we describe when and how the uni-system and the specific-FR-system are applied in different situations for successful primer design:

### uni-system

The uni-system is primarily used to design primers for miRNAs of interest when the miRNA is not a member of a family or when its sequence similarity is <90% of that compared to other miRNAs. The system utilizes the universal primer and a specific forward primer in most situations. The sequence of forward primer generally contains the same 12–18 nt of the 5′-end of the mature miRNA sequence. There are two designing steps in the uni-system to ensure the specificity of forward primer. First, an adjustment step is performed to ensure that the difference between the *T*_m_ value of the forward primer and the universal reverse primer does not exceed 2°C. In the case that the *T*_m_ value of the forward primer is <57°C, one or more nucleotide(s) from the 5′-tailed sequence is added sequentially to the 5′-end of the forward primer until a *T*_m_ of 59°C is achieved. Second, a confirmation step is performed to determine whether a self-dimer exists and/or hetero-dimer is formed between the forward primer and the universal reverse primer. Our methodology uses Δ*G*, a common characteristic used to assess the presence of dimers (Shen et al. 2010); when the Δ*G* of either the self-dimer or hetero-dimer is greater than −9.0 ± 1 kcal/mol, no additional modification is needed to be applied to the forward primer. In contrast, when the Δ*G* of the self-dimer or hetero-dimer is less than −9.0 ± 1 kcal/mol, either the 5′-tailed sequence is changed to other IUPAC alternatives or the dimer-forming nucleotides of the forward primer is removed. Both steps are iteratively performed until the conditions are satisfied. In several instances, it has been difficult to eliminate the dimer issue by designing and modifying only the forward primer, e.g., hsa-miR-9-5p. Therefore, it is recommended to modify both the forward primer and the reverse primer to avoid the dimer region. The process includes the reduction of the length of the 3′-end of the forward primer and the replacement of the universal reverse primer with a redesigned reverse primer. The sequence of the replacement reverse primer generally contains 2–8 nt residues complementary to the 3′-end of the mature miRNA sequence. By following the adjustment and confirmation steps, the difference in the *T*_m_ value of both primers should not exceed 2°C, thus eliminating the issue of primer dimer. If the *T*_m_ value of the replacement reverse primer is <55°C, adding at least one or more nucleotides to the 5′-end of the replacement reverse primer from the universal reverse sequence will increase the *T*_m_ value to 59°C. Finally, both steps are iteratively performed until all conditions are satisfied.

### specific-FR-system^overlap^

In most situations, primers have difficulties discriminating members of the miRNA families or similar miRNAs differed by a single-nucleotide or with sequence similarity >90%. Hence, the specific-FR-system is strongly suggested as the preferred process to increase the primer specificity and reduce the dimer issue. The specific-FR-system contains three sequential design rules/functions: (i) overlap, (ii) FPM, and (iii) RPM to address different problems. To begin with, the overlap method is required to perform a sequence comparison to determine the location(s) of nucleotide difference between members of miRNA families. The sequence of the forward primer generally contains at least the same 4-nt residues of the 5′-end of the mature miRNA sequence. The sequence of the reverse primer generally contains at least 4-nt residues complementary to the 3′-end of the mature miRNA sequence. Both the forward and the reverse primers are designed to overlap by a single nucleotide, which is the nucleotide that differs between miRNA family members. Similarly, there are two additional steps to ensure the specificity of the forward and reverse primers. First, an adjustment step is performed to ensure that the difference of the *T*_m_ value between the forward primer and the reverse primer does not exceed 2°C. In the case that the *T*_m_ value of the forward primer is <55°C, one or more nucleotide(s) from the 5′-tailed sequence is added sequentially to the 5′-end of the forward primer until a *T*_m_ of 59°C is achieved. Similarly, if the *T*_m_ value of the reverse primer is <55°C, one or more nucleotide(s) from the universal reverse sequence is added at the 5′-end of the reverse primer to increase the *T*_m_ value to 59°C. Secondly, a confirmation step is performed to determine whether a self-dimer exists and/or hetero-dimer is formed between the forward primer and the reverse primer. As mentioned above, Δ*G* is one of the common factors used to check for the presence of dimers; however, in this study, Δ*G* of less than −9.0 ± 1 kcal/mol usually causes problems in the qPCR reactions. If Δ*G* of either the self-dimer or hetero-dimer is greater than −9.0 ± 1 kcal/mol, the design of forward and reverse primers is completed. In contrast, when Δ*G* of either the self-dimer or hetero-dimer is less than −9.0 ± 1 kcal/mol, changing the 5′-tailed sequence to other IUPAC alternatives is able to reduce the formation of self-dimer or hetero-dimer. Both steps are iteratively performed until all conditions are satisfied. If the primer does not exhibit specificity after PCR assays, the number of overlapping nucleotides can be increased up to 2 nt. If the primer dimer continues to exist after performing all specific-FR-system^overlap^ modification steps, the specific-FR-system^FPM^ or specific-FR-system^RPM^ is then used to eliminate the issue.

### specific-FR-system^FPM^

If the dimer issue is caused by the reverse primer, the specific-FR-system^FPM^ is suggested to resolve the problem. The concept of the specific-FR-system^FPM^ is to eliminate the dimer-forming sequences of the reverse primer and enhance the specificity of the forward primer. The sequence of the forward primer is extended to where one of the nucleotide differences between the miRNAs is found. The sequence of the reverse primer is reduced from its 3′-end sequences but will need to contain at least 4-nt residues complementary to the mature miRNA sequences. If the dimer issue still cannot be resolved, the universal reverse primer is then used to replace the reverse primer. Finally, both adjustment and confirmation steps are iteratively performed until all conditions are met.

### specific-FR-system^RPM^

On the other hand, the RPM method is performed if the dimer issue is caused by the forward primer. Similarly to the specific-FR-system^FPM^, the specific-FR-system^RPM^ is used to resolve the formation of dimer region in the forward primer and improve the specificity of the reverse primer. The sequence of the reverse primer is extended to where one of the nucleotide differences is located. The sequence of the forward primer is trimmed from its 3′-end of sequence but will contain at least the same 4-nt residues of the mature miRNA sequences. Finally, both adjustment and confirmation steps are iteratively performed until all conditions are satisfied.

The designed primers are currently used on Quark Biosciences’ miRSCan line of PanelChip products, where multiple miRNAs can be analyzed on one array chip. The product numbers/names are PanelChip PCP-01 and PCP-02, and can be found on Quark Biosciences’ miRNA service catalog: http://download.quarkbiosciences.com/Brochure/miRNA_list.pdf

### Calculation of Δ*G* value

The Δ*G* value is calculated by RNAcofold algorithm using the DNA parameters (Gruber et al. 2008) and is set at 9.0 ± 1 kcal/mol. OligoAnalyzer 3.1 (Owczarzy et al. 2008) is the most frequently used tool to calculate the Δ*G* of a primer. Primers with a controversial Δ*G* value (e.g., see Table 2, hsa-miR-18b-5p-F with a Δ*G* −8.95 kcal/mol) are additionally confirmed by OligoAnalyzer 3.1 manually.

### Calculation of melting temperature

The *T*_m_ (melting temperature) value is calculated by the following formula:

$$T_{m}=\frac{1\text{E}3*H}{S+1.987*\log(C)-\text{log}(2\text{E}9)}-2.73.15,$$

where H and S denote the enthalpy and entropy, respectively, and *C* denotes the initial primer concentration (SantaLucia 1998; von Ahsen et al. 2001). In miPrimer, the difference of the *T*_m_ between the forward and the reverse primer should not exceed 2°C ± 0.1. OligoAnalyzer 3.1, is used to confirm the *T*_m_ manually as required.

### Calculation of cross-reactivity

The number of nucleotides that differ between each other is indicated in the matrix. Cross-reactivity between miRNA family members is shown as the mean of Cq and ΔCq (Cq_mm_ − Cq_pm_) in Quark Biosciences’ qPCR platform, where Cq_mm_ denotes Cq of miRNA against the heterologous primer and Cq_pm_ denotes Cq of miRNA against the homologous primer. ΔCq > 4 denotes that the cross-reactivity is <5% calculated by 10^ΔCq/S^ × 100%, where S is the slope of the standard curve (Wan et al. 2010).

### Calculation of false positive rate

False positive rate is calculated by *P*_NTC_/*N*, where *P*_NTC_ denotes the number of reactions in NTC assay, and *N* denotes the total number of qPCR reaction assays (in our case, 2500 reactions). In no-template control assays, any reaction will be treated as a false positive even if the Cq value is high.

### Synthetic miRNA and cDNA template

Synthetic single-stranded oligonucleotides, which contain the sequences of the universal RT primer ***5***′***-CAACTCAGGTCGTAGGCAATTCGTTTTTTTTTTTTTTTTTTTT-3***′, were designed to mimic the sequences of mature miRNA and miRNA cDNA and used as templates in our study. The oligonucleotides purchased from Protech Technology Enterprise Co., Ltd. or PURIGO Biotechnology Co., Ltd, were dissolved in TE buffer, aliquoted and stored at −80°C.

### Quantitative real-time PCR assay using DigiChip and Quark Biosciences’ qPCR platform

For qPCR efficiency assay, five 10-fold serial dilutions were made from the highest concentration (10^9^ copies/µL) by adding 10 µL of the previous solution to 90 µL nuclease-free H_2_O. Four microliters of each synthetic miRNA serial dilution was added to the qPCR mixtures containing 30 µL of 2× Quarkbio qPCR master mix (QuarkBiosciences, Inc.), 1.5 µL of 0.25 µM specific forward primer, and 1.5 µL of 0.25 µM specific reverse primer or universal reverse primer. Twenty-three microliters of nuclease-free water was added to the mixture to a final volume of 60 µL. The master mix was mixed thoroughly and briefly spun down to collect the liquid at the bottom. The master mix was then applied onto DigiChip. All reactions were run in triplicate.

For qPCR assays demonstrating that miRNA family members can be discriminated, 2 µL of the synthetic cDNA template (∼2 × 10^5^ copies) were added to the qPCR mixtures containing 30 µL of 2× Quarkbio qPCR master mix (QuarkBiosciences, Inc.), 1.5 µL of 0.25 µM specific forward primer, and 1.5 µL of 0.25 µM specific reverse primer or universal reverse primer. Twenty-five microliters of nuclease-free water was added to the mixture to a final volume of 60 µL. The master mix was mixed thoroughly, briefly spun down and then applied onto DigiChip.

DigiChip, a 36-mm × 36-mm × 1-mm reaction plate consisting of 2500 wells, was developed to be used with Quark Biosciences’ qPCR platform. To apply the qPCR mixture containing the template and the primer pairs to be tested, 60 µL of the mixture is dispensed using a pipetman along the edge of DigiChip. The qPCR mixture should cover more than the entire length of 50 wells along the edge. Fifty microliters of the qPCR mixture is then applied across the entire surface of the DigiChip via a scraping motion with a glass slide, resulting in 20 nL of master mix per reaction well. The DigiChip is then submerged, with reaction wells facing the bottom, into a tray containing mineral oil. Each plate is then placed into a thermal cycler for the amplification of the template. qPCR was performed according to the following cycling program for miRNA analysis: 95°C for 36 sec to denature for 1 cycle, followed by 95°C for 36 sec and 60°C for 72 sec for 40 cycles.

Quark Biosciences’ qPCR platform is a real-time quantitative/digital PCR Platform developed by Quark Biosciences, Inc. The thermal cycling functionality is accomplished by Thermal-Roller-Coaster, a proprietary technology which consists of six resistive heater blocks with different, but constant temperature. Amplification is achieved by shuttling DNA or cDNA samples between denaturing heater blocks and annealing/extension heater blocks. Each PCR run can accommodate up to six samples. The platform utilizes a white-light LED optics system with up to four different filter block formats to achieve illumination for samples with FAM, VIC, ROX, and Cy5 dye.

## SUPPLEMENTAL MATERIAL

Supplemental material is available for this article.

## Supplementary Material

Supplemental Material
