# Supplementary material for: miPrimer: an empirical-based qPCR primer design method for small noncoding microRNA
Source: RNA. 2018 Mar;24(3):304–12. doi: 10.1261/rna.061150.117 (PMC5824350; doi:10.1261/rna.061150.117)
Supplement: Supplemental Material [file supp_24_3_304__index.html]

miPrimer: an empirical-based qPCR primer design method for small noncoding microRNA — Supplemental Material 

# miPrimer: an empirical-based qPCR primer design method for small noncoding microRNA

## Supplemental Material

- Supplemental\_Material.docx
