# Supplementary material for: miPrimer: an empirical-based qPCR primer design method for small noncoding microRNA
Source: RNA. 2018 Mar;24(3):304–12. doi: 10.1261/rna.061150.117 (PMC5824350; doi:10.1261/rna.061150.117)
Supplement: Supplemental Material [file supp_061150.117_Supplemental_Material.docx]

# Supplementary Information

# Materials and Methods

**Synthetic cDNA template**

Synthetic single-stranded oligonucleotides, which contain the sequences of the universal RT primer ***5’-CAACTCAGGTCGTAGGCAATTCGTTTTTTTTTTTTTTTTTTTT-3’***, were designed to mimic the sequences of mature miRNA cDNA and used as templates in our study. The oligonucleotides purchased from Protech Technology Enterprise Co., Ltd. or PURIGO Biotechnology Co., Ltd, were dissolved in TE buffer, aliquoted and stored at -80℃.

**RNA sample**

Small RNAs were extracted from A2058 cell line using the NucleoSpin® miRNA kit (Macherey-Nagel) and stored at -80℃ until use.

**Reverse transcription**

5 x 10^6^ copies of synthetic hsa-let-7b miRNA (purchased from IDT) or 2 ng of small RNAs from A2058 cells were reverse transcribed in a 20-μl reaction using a universal RT primer with the poly(A)-based miRNA cDNA synthesis kit with (Quark Biosciences Inc.). cDNA was stored at -80°C until use.

**Quantitative real-time PCR assay using DigiChip^TM^ and Quark Biosciences’ qPCR Platform (Manuscript in preparation)**

For qPCR efficiency assay, five 10-fold serial dilutions were made from the highest concentration (10^9^ copies/μl) by adding 10 μl of the previous solution to 90 μl nuclease-free H_2_O. 4 μl of each synthetic miRNA serial dilution was added to the qPCR mixtures containing 30 μl of 2x Quarkbio qPCR master mix (Quark Biosciences, Inc.), 1.5 μl of 0.25 μM specific forward primer, and 1.5 μl of 0.25 μM specific reverse primer or universal reverse primer. 23 μl of nuclease-free water was added to the mixture to a final volume of 60 μl. The master mix was mixed thoroughly, and briefly spun down to collect the liquid at the bottom. The master mix was then apply onto DigiChip™. All reactions were run in triplicate.

For qPCR assay to discrimination of miRNA family, 2 μl of the synthetic cDNA template (~2*10^5^ copies) or 2 μl of RT product were added to the qPCR mixtures containing 30 μl of 2x Quarkbio qPCR master mix (Quark Biosciences, Inc.), 1.5 μl of 0.25 μM specific forward primer, and 1.5 μl of 0.25 μM specific reverse primer or universal reverse primer. 25 μl of nuclease-free water was added to the mixture to a final volume of 60 μl. The master mix was mixed thoroughly, and briefly spun down to collect the liquid at the bottom. The master mix was then apply onto DigiChip™.

DigiChip^TM^, a 36-mm x 36-mm x 1-mm reaction plate consisting of 2500 wells, was developed to be used with Quark Biosciences’ qPCR platform. To apply the qPCR mixture containing the template and the primers pairs to be tested, 60 μl of the mixture is dispensed using a pipetman along the edge of DigiChip^TM^. The qPCR mixture should cover more than the entire length of 50 wells along the edge. 50 μl of the qPCR mixture is then applied across the entire surface of the DigiChip^TM^ via a scraping motion with a glass slide, resulting in 20 nl of master mix per reaction well. The DigiChip^TM^ is then submerged, with reaction wells facing the bottom, into a tray containing mineral oil. Each plate is then placed into thermal cycler for the amplification of the template. qPCR was performed according to the following cycling program for miRNA analysis: 95°C for 36 seconds to denature for 1 cycle, followed by 95°C for 36 seconds and 60°C for 72 second for 40 cycles.

Quark Biosciences’ qPCR platform is a Real-time Quantitative/Digital PCR Platform developed by Quark Biosciences, Inc. The thermal cycling functionality is accomplished by Thermal-Roller-Coaster®, a proprietary technology which consists of six resistive heater blocks with different, but constant temperature. Amplification is achieved by shuttling DNA or cDNA samples between denaturing heater blocks and annealing/extension heater blocks. Each PCR run can accommodate up to 6 samples. The platform utilizes a white-light LED optics system with up to 4 different filter block formats to achieve illumination for samples with FAM^TM^, VIC®, ROX^TM^, and Cy5^TM^ dye.

**Quantitative real-time PCR assay using** **Bio-Rad CFX96™ system**

For qPCR efficiency assay, five 10-fold serial dilutions were made from the highest concentration (10^9^ copies/μl) by adding 10 μl of the previous solution to 90 μl nuclease-free H_2_O. 1.33 μl of each synthetic miRNA serial dilution (the same concentration of template with DigiChip^TM^) was added to the qPCR mixtures containing 10 μl of 2x Quarkbio qPCR master mix (Quark Biosciences, Inc.), 0.5 μl of 0.25 μM specific forward primer, and 0.5 μl of 0.25 μM specific reverse primer or universal reverse primer. Nuclease-free water was added to the mixture to a final volume of 20 μl. qPCR analysis was performed on the Bio-Rad CFX96™ system according to the following cycling program for miRNA analysis: 95°C for 3 min to denature for 1 cycle, followed by 95°C for 10 seconds and 60°C for 30 second for 40 cycles. All reactions were run in triplicate. RT-qPCR of the synthesized cDNA from cell line A2058 followed the above protocol. 0.2 ng of cDNA was used as the template.

# Figures Legends

**Figures S1. qPCR efficiency of miRNA primer designed by miPrimer™ and performed by Quark Biosciences’ qPCR Platform.**

(A) qPCR amplification curves of a serially diluted oligonucleotide hsa-miR-122-5p miRNA template against hsa-miR-122-5p-F primer and universal reverse primer designed by ***uni-system*** (four 10-fold dilutions; 2500 qPCR reactions per dilution). (B) qPCR amplification curves of a serially diluted synthetic oligonucleotide hsa-miR-10a-5p against hsa-miR-10a-5p-F/R designed by ***specific-FR-system^FPM^*** (four 10-fold dilutions; 2500 qPCR reactions per dilution). Each dot represents mean Cq ± SD from three replicates. The List of miRNA primer sequences was shown in the Table S1.

**Figures S2. qPCR efficiency of five miRNA primer sets designed by miPrimer™ and performed by Bio-Rad CFX96™ system.**

qPCR amplification curves of a serially diluted oligonucleotide (four 10-fold dilutions; 2500 qPCR reactions per dilution). (A) hsa-miR-9-5p miRNA template against hsa-miR-9-5p-F primer and universal reverse primer, (B) hsa-miR-122-5p miRNA template against hsa-miR-122-5p-F primer and universal reverse primer, (C) hsa-let-7b-5p against hsa-let-7b-5p-F/R, (D) hsa-miR-10a-5p against hsa-miR-10a-5p-F/R, and (E) artificial template against artificial template-F and universal reverse primer. Primer sets of (A), (B), and (E) were designed by ***uni-system***, and primer sets of (C) and (D) were designed by ***specific-FR-system^FPM^*** . Each dot represents mean Cq ± SD from three replicates.

# Supplementary Figures and Tables

**Figures S1. qPCR efficiency of miRNA primer sets designed by miPrimer™ and performed by Quark Biosciences’ qPCR Platform.**


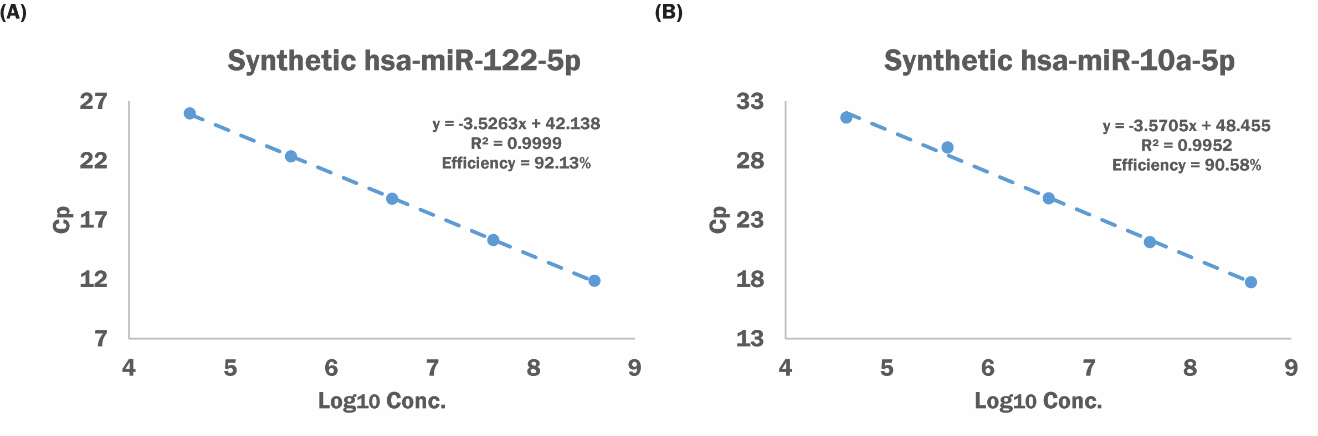


**Figures S2. qPCR efficiency of miRNA primer designed by miPrimer™ and performed by Bio-Rad CFX96™ system.**


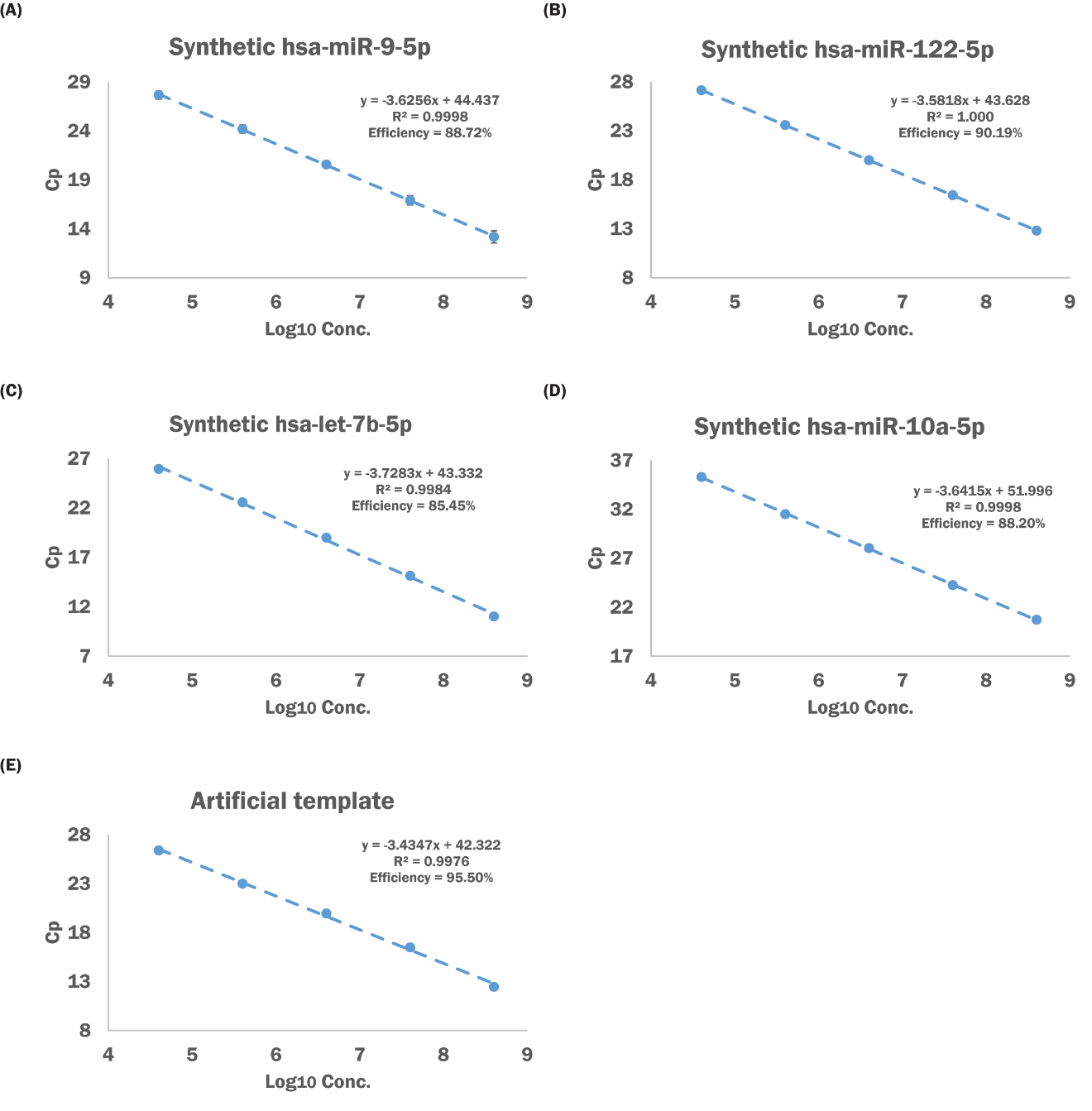
 **Table S1. List of miRNA primer sequences used for qPCR analysis in this study**

**
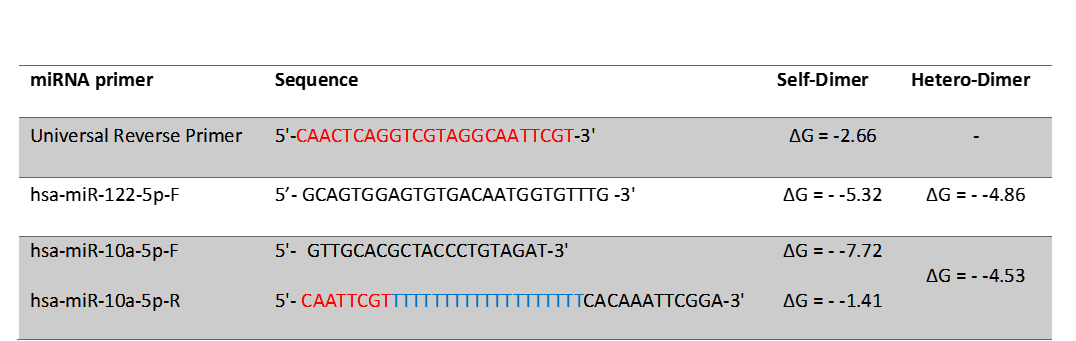
**

**Table S2. Discrimination among the closely related members of the let-7 family by using the let-7b specific primer pair designed by miPrimer™.**

**
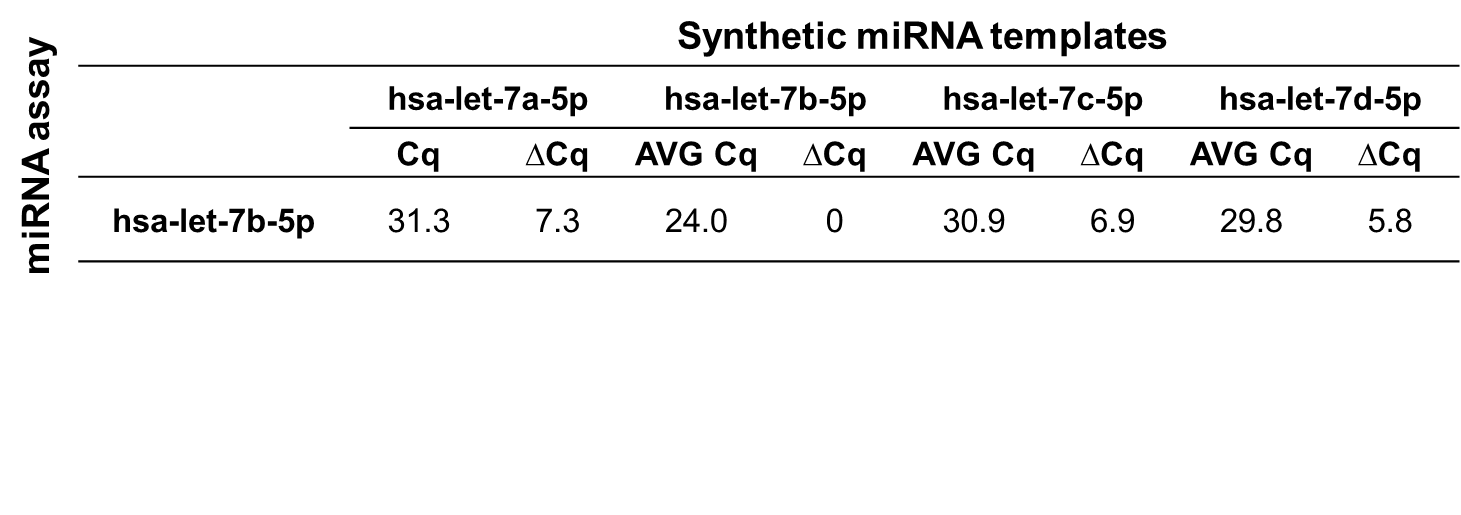
** Note: Reverse transcription was performed using synthetic miRNA templates followed by qPCR reaction.

**Table S3. Primer sets designed for seven miRNAs were detected in A2058 cells using Bio-Rad CFX96™ system, where each set exhibit only one peak (single melting temperature), suggesting the primer sets only recognized their respective target.**

Note: RT-qPCR analysis was performed using Bio-Rad CFX96™ system for seven pairs of miPrimer™-designed primer sets targeting different miRNAs. Two technical replicates were performed for each miRNA and the resulting Cq and melting temperature of each miRNA were shown in the Table. In addition to the observed single peak, no signal was detected in the NTC (no-template control) reactions. Rep: Replicate, NTC: no-template control, ND: not detected.
